# Research Progress on the Regulating Factors of Muscle Fiber Heterogeneity in Livestock: A Review

**DOI:** 10.3390/ani14152225

**Published:** 2024-07-31

**Authors:** Yufei Wang, Donghao Zhang, Yiping Liu

**Affiliations:** State Key Laboratory of Swine and Poultry Breeding Industry, Key Laboratory of Livestock and Poultry Multi-Omics, Ministry of Agriculture and Rural Affairs, and Farm Animal Genetic Resources Exploration and Innovation Key Laboratory of Sichuan Province, College of Animal Science and Technology, Sichuan Agricultural University, Chengdu 611134, China; wyfff@stu.sicau.edu.cn (Y.W.); dhzhang934@163.com (D.Z.)

**Keywords:** muscle fiber type, meat quality, livestock

## Abstract

**Simple Summary:**

Skeletal muscle fibers, the smallest functional unit of skeletal muscle, directly affect the normal growth and development of animal muscles and the quality of carcass muscle after slaughter. Individual characteristics, feeding, and genetic factors influence the heterogeneity of muscle fibers in livestock. Notably, genetic factors rapidly and efficiently improve the proportion and conversion of animal muscle fibers with the development of omics technology and sequencing technology. Therefore, only a global approach including all parameters seems to be pertinent to understand the relationships among the characteristics of myofibers, growth performance, and meat quality.

**Abstract:**

The type of muscle fiber plays a crucial role in the growth, development, and dynamic plasticity of animals’ skeletal muscle. Additionally, it is a primary determinant of the quality of both fresh and processed meat. Therefore, understanding the regulatory factors that contribute to muscle fibers’ heterogeneity is of paramount importance. Recent advances in sequencing and omics technologies have enabled comprehensive cross-verification of research on the factors affecting the types of muscle fiber across multiple levels, including the genome, transcriptome, proteome, and metabolome. These advancements have facilitated deeper exploration into the related biological questions. This review focused on the impact of individual characteristics, feeding patterns, and genetic regulation on the proportion and interconversion of different muscle fibers. The findings indicated that individual characteristics and feeding patterns significantly influence the type of muscle fiber, which can effectively enhance the type and distribution of muscle fibers in livestock. Furthermore, non-coding RNA, genes and signaling pathways between complicated regulatory mechanisms and interactions have a certain degree of impact on muscle fibers’ heterogeneity. This, in turn, changes muscle fiber profile in living animals through genetic selection or environmental factors, and has the potential to modulate the quality of fresh meat. Collectively, we briefly reviewed the structure of skeletal muscle tissue and then attempted to review the inevitable connection between the quality of fresh meat and the type of muscle fiber, with particular attention to potential events involved in regulating muscle fibers’ heterogeneity.

## 1. Introduction

In recent decades, global consumption of meat protein has increased in parallel with rising per capita income, leading to the development of and improvement in dietary structures [1,2]. Modern breeding strategies aim to enhance the growth rate, feed conversion efficiency, and the percentage of lean meat in livestock and poultry, thereby producing large-scale commercial meat to meet consumer demand. However, this approach, which focuses on rapid meat production, has led to a decline in meat quality despite improving the production efficiency of animal husbandry [3,4]. Meat quality is a critical factor influencing consumers’ purchasing decisions, with the morphological characteristics and functional properties of skeletal muscle fibers playing a pivotal role [5].

Animal muscle fibers, primarily composed of long, fine myofibrils organized in parallel, can be considered to be cylindrical cells with multiple nuclei [6]. Myosin heavy chain isomers enable the classification of muscle fibers into four categories: Type I slow oxidative, Type IIA fast oxidative, Type IIX transitional or oxidative/glycolytic, and Type IIB fast glycolytic. Type IIX fibers, a distinct third type of fast muscle fiber, were later identified through studies using monoclonal antibodies against MyHC and improved electrophoresis techniques. Functionally, Type IIX fibers exhibit characteristics and fatigue resistance that fall between those of Type IIA and Type IIB fibers. Although types of muscle fiber are fixed during the embryonic stage, there is a postnatal transformation between different types of muscle fiber (I ↔ IIA ↔ IIX ↔ IIB) that adapts the muscle to the body’s dynamic demands [7]. The biochemical and structural characteristics of muscle fibers intrinsically influence various indicators of meat quality, including color, tenderness, flavor, water retention capacity, and post-slaughter pH level [8]. Notably, increasing the proportion of Type I muscle fibers in muscle can effectively enhance the color of fresh meat.

Several factors can impact the type of muscle fiber, including breed, age, sex, nutrition, environmental temperature, hormones, the muscles’ location, and molecular genetics [9]. An integrated analysis of these internal and external factors related to muscle fibers’ heterogeneity can provide the theoretical basis necessary for producing high-quality meat products. This article reviews the factors influencing muscle fibers’ distribution and transformation in livestock, with a particular focus on the influence of molecular genetic mechanisms on muscle fibers’ heterogeneity. Elucidating the fiber type-specific mechanisms controlling growth will help us understand how heterogeneous tissues respond to changing environments and markets.

### 1.1. Skeletal Muscle Fibers

Today, global demand for meat products is rising, particularly in developing countries [10,11]. Meat products consumed by consumers represent approximately 35% to 60% of the body weight of slaughtered livestock and poultry. As a major component of meat products, the growth and development of skeletal muscle directly impact the quantity and quality of these products [9,12,13]. Mammalian skeletal muscle is primarily composed of multinucleated muscle fibers, with smaller amounts of connective and adipose tissue [14,15]. Mature multinucleated muscle fibers, the smallest functional units of skeletal muscle, are formed during embryonic development and possess a degree of excitability and contractility [9,16]. Skeletal muscle continually remodels in response to different metabolic processes (oxidative or glycolytic) and functional demands (rapid or slow contraction), due to the highly heterogeneous distribution of multinucleated myofibers within it [17,18]. Additionally, rat primary fibers form Type I slow-oxidizing, fatigue-resistant red muscle, while secondary and tertiary fibers form Type II fast-glycolytic, fatigue-resistant white muscle [19,20,21].

### 1.2. Classification Methods of Skeletal Muscle Fiber 

**Figure 1:**
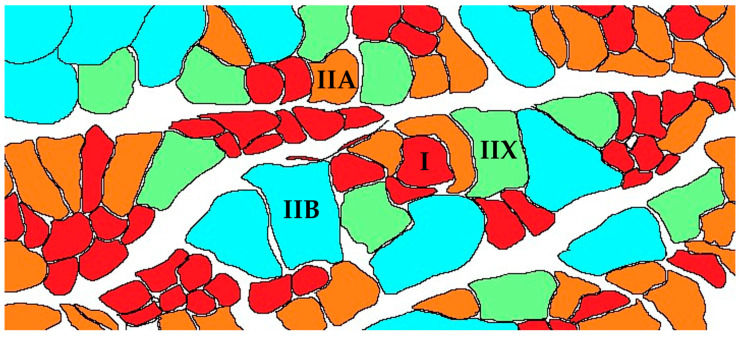
Diagram of types of muscle fibers.

In 1874, Ranvier conducted the initial study on skeletal muscle fibers in cats, categorizing them into slow and fast fibers based on their twitching speed. This early work laid the foundation for understanding the types of muscle fiber. Later, in 1960, Buller and Eccles discovered that muscle fibers can transform their contractile properties, further advancing research into the transformation of the types of mammalian skeletal muscle fibers [22,23]. Currently, there are numerous classification criteria for the types of muscle fiber, including color, characteristics of muscle contraction under electrical stimulation, single twitch speed, fatigue resistance upon continuous activation, histochemical staining, predominant enzyme pathways, and the expression of myosin heavy chain (MyHC) isoforms. In this review, mammalian skeletal muscle fibers were categorized into four types: I, IIA, IIX, and IIB, primarily on the basis of the expression patterns of MyHC isoforms (Figure 1) [24,25,26].

Myosin is an active protein with ATPase activity that is crucial for muscles’ twitching and contraction [27]. Myosin II, also known as conventional myosin, was the first isoform isolated from muscle tissue’s thick and thin filaments [28]. This molecular motor extends its ATP-bound “Y”-shaped head to interact with actin, forming a cross-bridge that is essential for mechanical movement [29]. Specifically, the genes *MYH7*, *MYH2*, *MYH1*, and *MYH4* encode MyHC Types I, IIA, IIX, and IIB, respectively [18].

The types of muscle fibers—I, IIA, IIX, and IIB—are found in rodents and the majority of mammalian species [30]. Rodents serve as crucial model organisms for studying the growth and development of skeletal muscle due to their ability to divide muscle groups into either Type I or Type II muscle fibers [31]. In human skeletal muscle, the predominant fiber types are Type I, Type IIA, and Type IIX, distributed fairly evenly throughout the body [32,33]. Notably, research by Stefano Schiaffino using single-fiber proteomic profiles demonstrated that Type IIB muscle fibers are present only in trace amounts in human leg muscles [34]. Livestock also exhibit all these types of muscle fiber (Type I, Type IIA, Type IIX, and Type IIB). Different livestock exhibit various types of muscle fiber. For instance, pigs and cattle possess Type I, IIA, IIX, and IIB fibers. However, it is challenging to differentiate between Type IIB and Type IIX fibers in cattle using conventional chemical techniques. Compared with other large meat-producing livestock, poultry skeletal muscles contain relatively fewer oxidative fibers. Specifically, Type IIB fibers are primarily found in the pectoralis major muscle, while Type I, IIA, and IIX fibers may be present in the leg muscles [35]. Additionally, the classification of the types of muscle fibers in poultry exhibits unique characteristics, notably with Type IIX(D) fibers being rare [35]. The distribution of the types of muscle fibers in chickens is particularly species-specific, with nearly all studies consistently indicating that the pectoralis major muscle in chickens comprises fast-twitch fibers exclusively expressing glycolytic fibers. Some research has also identified other categories, such as the Type IIIA and IIIB multi-tension nerve-regulated slow fibers unique to avian muscles, which are located in the latissimus dorsi anterior, deep adductor, and plantar muscles [36]. However, due to the significant inherent genetic differences between mammals and poultry, there is currently a lack of systematic studies on the differences in the composition of types of muscle fibers, muscle fibers’ characteristics, and quality traits among different livestock species.

The contraction speed of muscle fibers is influenced by the level of Ca^2+^-activated ATPase activity in MyHC isoforms, with Type I fibers exhibiting the lowest activity, followed by Type IIA, Type IIX, and Type IIB, which has the highest activity [37,38]. Type I muscle fibers are classified as slow-twitch, characterized by oxidative metabolism, high endurance, and fatigue resistance [7]. Type IIA fibers are intermediate, capable of rapid contraction, and primarily oxidative but also possess glycolytic capacity [39]. In contrast, Type IIX and IIB fibers are fast-twitch fibers, relying predominantly on glycolytic metabolism, less resistant to fatigue, and depleting glycogen stores quickly [40]. (See Table 1).

## 2. Effect of the Composition of Types of Muscle Fibers on Meat Quality

The term “meat” refers to the edible muscle and visceral parts of livestock and poultry after the removal of most adipose and connective tissues [41]. Meat products are crucial sources of essential nutrients and are widely appreciated for their distinctive flavors [42,43]. As living standards improve globally, consumers increasingly demand high-quality fresh meat products and have higher standards for their purchases [44], particularly favoring red meat [45]. Evaluating meat quality involves multiple criteria including biochemical characteristics, sensory perceptions, animal welfare, health standards, and other relevant factors [46]. In a narrower sense, “meat quality” focuses on sensory factors such as appearance, flavor, tenderness, and color, as well as other important attributes such as water content and pH, which influence these sensory factors [47,48], though a universally accepted synthesis of the evaluation criteria remains lacking.

The diverse composition of muscle fibers and their proportions in the carcass significantly influence muscles’ metabolism and, consequently, meat quality [5,49]. Meat color, a key factor in consumers’ perception, is largely determined by the myoglobin content [47]. Bright red meat results from a higher proportion of oxidative muscle fibers such as Types I and IIA, while less red meat typically contains more glycolytic Type IIB fibers. Additionally, the type and size of muscle fibers can influence the flavor of meat. Generally, finer muscle fibers are associated with enhanced flavor due to their higher surface area and potential for greater interaction with flavor compounds (Type I < Type IIA < Type IIX < Type IIB) [50]. Specifically, oxidative slow muscle fibers (Type I) contain higher levels of umami amino acids such as glutamate and inosinate, which contribute to the savory taste of processed meats. This is supported by studies showing that these fibers are rich in myoglobin and have higher mitochondrial density, leading to a greater presence of compounds that enhance the umami flavor [51]. Thus, while the relationship between the type of muscle fiber and the flavor of meat involves both objective measures (e.g., amino acid content) and subjective sensory evaluations, oxidative slow fibers play a significant role in determining the savory characteristics of meat. Conversely, glycolytic fast muscle fibers have more glycolytic enzymes and sarcoplasmic reticulum, leading to anaerobic metabolites post-slaughter, potentially resulting in pale, soft, exudative (PSE), or red, soft, exudative (RSE) meat textures [52,53].

The distribution of types of muscle fibers in various animals has been observed to correlate with the final quality of the meat product. For instance, in Jeju black pigs (JBPs), Type I slow muscle fibers show a positive correlation with pH_45_ value and tenderness, while correlating negatively with luminance and drip loss. Conversely, Type IIB fast muscle fibers correlate positively with luminance and drip loss, but negatively with pH_45_ values [54,55]. Peking duck and Liancheng White duck have higher proportions of Type I muscle fibers, which positively correlate with pH_24_ values, water-holding capacity, and intramuscular fat content, and negatively correlate with shear stress [56]. Xueshan chickens, known for the larger cross-sectional area of their chest muscle and the higher proportion of oxidative muscle fibers, exhibit increased muscle redness and intramuscular fat content [57]. As animals age, changes in the composition of muscle fibers affect the meat quality. Yangzhou geese undergo a transformation from Type II to Type I muscle fibers with advancing age, leading to increased meat redness, luminance, protein content, and intramuscular fat [58]. Korean native cattle show a positive correlation between Type I and Type IIA oxidative muscle fibers and traits of meat quality such as water-holding capacity, marbling, meat color, and tenderness. Conversely, Type IIX and Type IIB glycolytic muscle fibers exhibit a negative correlation with these traits [59,60]. In the infraspinatus muscles of yak, predominantly composed of Type I muscle fibers, correlations include a positive relationship with pH, initial H_2_O_2_, myoglobin, and antioxidant enzymes, and a negative correlation with lipid peroxidation, metmyoglobin, and lactate dehydrogenase activity [61]. Notably, an increased proportion of Type I muscle fibers is beneficial for reducing oxidative losses during meat processing, highlighting their role in maintaining meat quality [61,62,63].

In fact, the quality of fresh meat is largely determined by consumers’ preferences. However, the composition of the types of muscle fibers has a significant impact on the appearance, dietary quality, and trustworthiness of meat, indicating that characteristics of muscle fibers fundamentally influence meat quality. Specifically, oxidative muscle fibers (e.g., Type I and IIA) are positively correlated with higher tenderness, intramuscular fat, water-holding capacity, brightness, and color, whereas glycolytic muscle fibers (e.g., Type IIX and IIB) are associated with higher shear force and brightness [64,65,66]. Additionally, the high myoglobin content and flavor-enhancing amino acids in Type I muscle fibers particularly enhance meat’s color and flavor [67,68,69]. Therefore, controlling the quality of fresh meat through the characteristics of muscle fiber may be the best approach for progressively improving meat quality.

## 3. Potential Factors Influencing Myofibers’ Heterogeneity

### 3.1. Individual Characteristics

#### 3.1.1. Breed

The objective of modern breeding programs is to enhance the productivity of livestock, leading to the development of commercial breeds such as pigs, chickens, and cattle optimized for rapid growth and efficient meat production [70]. However, alongside improving production efficiency, it is crucial to maintain consistent high-quality meat production standards [71]. Variations in the geographical environment, breeding objectives, and methods contribute to significant differences in the characteristics of muscles between Chinese and foreign livestock and poultry breeds, particularly in the composition of skeletal muscle fibers.

Chinese local pig breeds often exhibit a higher proportion of Type I oxidized muscle fibers and oxidative enzyme activities, contributing to substantial intramuscular fat and reduced drip loss. In contrast, Western commercial pig breeds tend to have a higher prevalence of Type II glycolytic muscle fibers, which can result in diminished cooking taste and flavor [72,73]. Chinese Laiwu pigs exhibit more Type I slow muscle fibers and fewer Type II fast muscle fibers compared with DLY pigs, positively correlated to the intramuscular fat content and taste of the meat [74]. Additionally, Chinese Lantang pigs have been observed to express higher mRNA levels of MyHC-I and MyHC-IIA at birth compared with landrace pigs, while the expression of MyHC-IIB is lower [75].

Broiler chickens, bred for rapid growth, exhibit increased diameters and numbers of muscle fibers, with Type II muscle fibers predominating and increasing with age [76,77]. Comparatively, Arbor Acres broilers show a shift from red to white muscle fibers over time, reflecting the conversion of muscle fiber and altered energy metabolism (Type I ↔ Type II) [78]. Major fast-growing broiler strains such as Ross 308 and Cobb 500 are characterized by a predominance of Type II fast muscle fibers, facilitating rapid muscle development and a high protein content. In contrast, local slow-growing broilers tend to have higher proportions of oxidative muscle fibers and intramuscular fat, resulting in superior meat quality and color compared with fast-growing breeds [57,79]. In Pekin ducks, breed differences are evident in the characteristics of muscle fibers, with Cherry Valley Pekin ducks showing larger muscle fiber diameters and reduced fiber density, potentially affecting the intramuscular fat content [80,81]. Further studies are required to fully understand how these breed-specific differences impact the composition and quality of types of muscle fibers.

In the period before birth, Holstein Friesian dairy, German Angus beef, Galloway beef, and double-muscled Belgian Blue beef show no significant differences in the development of the number of muscle fiber. However, postnatally, double-muscled Belgian Blue cattle have been observed to possess a greater total number of muscle fibers compared with other breeds. Specifically, they exhibit a higher proportion of Type IIB glycolytic muscle fibers relative to Type I and Type IIA oxidative muscle fibers [82,83,84]. Dairy cattle, in contrast to beef cattle, typically have a higher prevalence of oxidized muscle fibers and greater oxidase activities, reflecting reduced reliance on glycolytic metabolism in muscle tissue [85]. In hybrids such as F1 Simmental × Jinjiang yellow cattle, there is an observed increase in the mRNA expression of MyHC-I in highrib and ribeye muscles compared with local Jinjiang yellow cattle, while the expression of MyHC-II decreases [86]. Piemontese cattle, compared with Belgian Blue bulls, show a lower number of Type IIA oxidative fast muscle fibers and a higher number of Type IIX glycolytic fast muscle fibers [87]. Texel sheep are characterized by Type II white muscle fibers, whereas Scottish Blackface sheep exhibit the characteristics of Type I red muscles [88]. In Suffolk ram × Finnish Landrace–Southdown ewes, α-W muscle fibers (Type IIB catabolism) predominate, but there are more β-R muscle fibers (Type I oxidation) compared with Suffolk ram × Suffolk–Rambouillet ewes. These breed differences may lead to the type of muscle fibers switching from α-R (Type IIX) to α-W (Type IIB) [88,89].

With the rapid development of the livestock industry to meet the dynamic demands of consumers, it is crucial to have a fundamental understanding of the intervention strategies that influence the growth of muscles and meat quality. This understanding is essential to improve production efficiency while maintaining a balance of scientific, economic, and environmental considerations to ensure the sustainability of meat production. Additionally, due to differences in geographical environments, breeding objectives, and husbandry practices, the characteristics of the muscles in livestock and poultry breeds vary significantly worldwide, especially in terms of the composition of skeletal muscle fiber. Therefore, focusing on breed factors that affect the transformation of muscle fibers ultimately points to the comprehensive influence of general factors.

#### 3.1.2. Age

Different types of muscle fiber have varying effects on the energy metabolism of carcass muscles, and age can influence the composition of these types of muscle fiber, interacting with one another. Optimizing the marketing age of livestock and poultry is beneficial for the production of high-quality meat. This is achieved by increasing the proportion of Type I oxidative muscle fibers, reducing the proportion of Type IIB glycolytic muscle fibers, and promoting their conversion to Type IIX muscle fibers [8,90]. Fei et al. increased slow muscle fibers (Type I), decreased fast muscle fibers (Type IIB), and promoted the conversion of muscle fibers (fast → slow) by building transgenic pigs overexpressing *PGC-1α* [91]. Bo Wang et al. added Vitamin A to calves’ diets, increased oxidative muscle fibers (Type I and Type IIA), decreased glycolytic muscle fibers (Type IIX), and promoted the muscle fibers to convert to oxidative types [92].

From birth until the eighth week postpartum, the proportion of Type I slow muscle fibers in the M. flexor digitorum superficialis, M. vastus intermedius, and soleus of pigs continues to increase, while the fast muscle fibers in the hind limb muscles differentiate into Type IIA oxidative muscles and Type IIB glycolytic muscles [93,94]. From the late embryonic stage through to the early neonatal stage, the mRNA expression of MyHC-IIB in pig muscle is significantly upregulated, indicating a high degree of dynamic plasticity in muscle fibers. Regulating these types of fibers during this developmental stage may be an effective means of improving pork quality [95]. In Ningxiang pigs, the mRNA expression levels of MyHC-I and MyHC-IIX initially decline and then increase with the onset of feeding, whereas the mRNA expression level of MyHC-IIA shows the opposite pattern, initially increasing and then declining before reaching a new peak [96]. At birth, the number of Type I, IIA, and IIX muscle fibers is greater in Chinese Lantang and Landrace pigs, while the number of Type IIB muscle fibers are greater at 90 days postpartum [75].

Smith et al. observed that the diameter of muscle fibers increased with age in broilers, but age at rearing did not seem to influence the type of muscle fiber [97]. Conversely, Y. No et al. found that with advancing age, the Type IIB muscle fibers in the caudal iliotibialis lateralis declined (from a 4:1 to a 1:1 ratio), while the Type I and Type IIA oxidative muscle fibers in the femorotibialis medius showed a gradual increase [98]. Shu et al. reported an increase in the number of Type I muscle fibers and a decrease in Type IIB muscle fibers in the leg muscles of Gaoyou ducks from Day 21 of the late embryonic period to the early stages after birth [99]. In Muscovy ducks, the cross-sectional area of Type IIA muscle fibers increased with age, while the cross-sectional area of Type IIB muscle fibers increased only between 8 and 10 weeks of age. However, the impact of feeding age on the distribution of muscle fibers in Muscovy ducks remains unclear [100]. Weng et al. observed that the types of muscle fiber in goose breast and leg muscles were not influenced by age at feeding, and they remained predominantly composed of Type IIA oxidative and Type IIB glycolytic fibers [58]. They noted, however, that muscles’ redness, muscle fibers’ cross-sectional area, protein content, intramuscular fat content, and shear force increased.

Picard et al. studied Montbéliard calves (2–6 months of age) and observed a shift from Type IIA to Type IIB muscle fibers in the semitendinosus and longissimus thoracis muscles, accompanied by a shift in muscle metabolism towards a glycolytic phenotype [101]. In Salers and Limousin cattle, the diameter of muscle fibers and the number of Type IIA fibers increased, while the number of Type IIB fibers decreased from 10 to 16 months of age [102,103]. Additionally, older bulls were more likely to develop atrophy of Type II muscle fibers. Hwang et al. found that adult goats had a considerably higher density of Type IIB fibers in their muscles compared with juvenile goats [104]. However, the results indicated that longer rearing days increased the muscles’ shear force. In sheep, the percentage of Type II muscle fibers in the medial-lateral femoral and rectus femoral muscles decreased with increased rearing age (from 85% to 72%) [105]. In horses, Type I and IIA muscle fibers, as well as the muscles’ oxidative metabolism, increased with age, while Type IIB muscle fibers decreased [106].

#### 3.1.3. Sex

In modern breeding, sex determines an animal’s value, particularly in laying hens, dairy cows, gestating sows, and similar animals. The current literature primarily reports sex differences in the composition, type, and size of human skeletal muscle fibers [107]. Sexual dimorphism has been observed in the masseter muscle of domestic rabbits and Muscovy ducks. For instance, the masseter muscle of male rabbits comprises 80% Type IIA muscle fibers, with Type I muscle fibers converting to Type IIA, while female rabbits have primarily Type I muscle fibers [108,109]. Notably, the mRNA expression of MyHC-IIA decreases, and the mRNA expression of MyHC-IIX increases in rabbit masseter muscle fibers following testosterone treatment [110]. Baéza et al. noted significant sexual dimorphism in Muscovy ducks, primarily reflected in differences in body size. This difference may be due to the larger cross-sectional area of muscle fibers or the higher total number of muscle fibers in male Muscovy ducks. However, the study did not indicate the effect of sex on muscle fibers’ heterogeneity [111].

In both piglets and finishers, sex does not the composition of affect muscle fibers but influences the cross-sectional area, with boars having muscle fibers with larger diameters [112,113]. Castrated boars were found to have more primary muscle fibers than sows, although there was no significant difference in the number of secondary muscle fibers. Shoko Sawano et al. observed that the proportion of Type I muscle fibers in castrated boars and sows was similar, but gilts showed a slight increase in the proportion of MyHC-I [114]. For commercial broilers, there is no evidence that sex affects the composition of the types of muscle fibers [70]. However, in Silkie chickens, roosters have significantly more Type IIR (red) muscle fibers than hens [115]. Compared with castrated bulls, intact bulls have more glycolytic muscle fibers, which facilitate the production of leaner meat [85]. Arnold et al. showed no significant difference in the proportions of types of muscle fiber among rams, wethers, and ewes [116]. There is limited literature on whether sex affects the composition of types of muscle fibers in livestock and poultry, indicating a need for further research to clarify the relationship between sex and the composition of muscle fibers.

#### 3.1.4. Muscle Area

The types of muscle fiber present in vertebrate skeletal muscles exhibit regional variations. Typically, the proportion of oxidized muscle fibers is higher in deep muscles compared with superficial muscles. Muscle fibers can transform into each other to meet the body’s requirements of energy metabolism [117,118]. Generally, different types of muscle fibers in an animal have distinct metabolic and contractile properties, and function in specific areas of the muscle [119].

LeMaster et al. found that the carcasses of slaughtered sows had a higher proportion of Type IIB glycolytic muscle fibers in the cutaneous trunci. In contrast, the masseter and psoas major muscles exhibited a greater proportion of Type I and Type IIA oxidative muscle fibers and a lower proportion of Type IIB glycolytic muscle fibers [53]. In Huai pig muscle tissue, the longissimus dorsi and biceps femoris were predominantly composed of fast-twitch glycolytic fibers, while the psoas major contained oxidized and intermediate fibers [73]. Kim et al. correlated the expression of muscle protein with the distribution of muscle fibers, revealing that oxidative muscle fibers were predominantly located in the dark portion of the muscle, while glycolytic fibers were primarily in the brighter region, significantly impacting the quality of pork [120].

Sakakibara et al. studied the muscle composition of Silkie chickens and White Leghorn chickens, finding that the M. iliotibialis lateralis was predominantly composed of IIR muscle fibers (red), whereas the M. pectoralis exhibited a predominance of IIW muscle fibers (white) or a minimal presence of IIR muscle fibers (<1%) [115]. In Chinese Qingyuan partridge chickens, the pectoralis major muscle is primarily composed of glycolytic muscle fibers, while the sartorius muscle is primarily composed of oxidized muscle fibers [121]. In a chicken model of liver fibrosis, a reduction in the number of Type II muscle fibers in the pectoral muscle was observed, while the number of Type I fibers in the lateral tibia increased [122]. This variation in the muscle fibers’ response to hepatic fibrosis is contingent upon the specific type of muscle fiber. Additionally, in broiler pectoralis major muscles, Type IIB fibers had small, poorly interconnected mitochondria, whereas Type I fibers in the gastrocnemius muscles had elongated, numerous mitochondria forming extensive networks [123].

In bulls, the semitendinosus is primarily composed of Type IIA oxidizing fast muscle fibers and Type II glycolytic fast muscle fibers, while the longissimus dorsi consists mainly of Type I slow muscle fibers, engaging in oxidative metabolism [85,124]. Marie et al. observed that Charolais cattle had a higher proportion of Type I oxidized red muscle fibers in the rectus abdominis compared with the triceps brachii and longissimus thoracis, with a lower proportion of Type IIX glycolytic white muscle and Type IIA oxidized white muscle [125]. In yaks, the biceps femoris is primarily composed of oxidized muscle fibers, while the obliquus externus abdominis is mainly composed of glycolytic muscle fibers [126]. Toshihiro et al. found that in sheep, Type I fibers were predominantly distributed in the tibialis cranialis muscle, soleus muscle, and flexor digitorum superficialis muscle. Additionally, the deep portion of the flexor digitorum superficialis exhibited a higher concentration of Type I fibers, comprising over 90% of the total muscle fiber population [127,128]. Mongolian horses exhibit a greater number of fast muscle fibers in the gluteus medius muscle and more oxidized slow muscle fibers in the splenius muscle [129]. Furthermore, the types and proportions of muscle fibers in different muscle groups of rabbits vary; for example, the tibialis anterior muscle predominantly consists of Type II fibers, while the extensor digitorum longus muscle is primarily constituted of Type I fibers [130].

### 3.2. Feeding Patterns

#### 3.2.1. Nutritional Regulation

The addition of specific nutrients or alterations in nutrient levels during livestock and poultry breeding can significantly influence the growth and development of animal skeletal muscle, leading to changes in the types of muscle fiber and thereby regulating the quality of fresh meat [131,132,133]. Notably, the biogenesis of mitochondria can influence the oxidative capacity of muscle and the number of oxidized muscle fibers. Additionally, maternal nutrition and health during pregnancy plays a pivotal role in the formation and conversion of muscle fibers in the offspring.

L-theanine, also known as N-ethyl-γ-glutamine, is a non-protein-derived amino acid that constitutes 60% to 70% of the total free amino acid content in tea [134]. Dietary L-theanine supplementation has been found to enhance the proportion of Type I muscle fibers in the skeletal muscle of both piglets and fattening pigs, facilitating the transformation of Type II glycolytic fast muscle into Type I oxidizing slow muscle [135,136]. Supplementing broilers’ diets with 600–900 mg/kg of L-theanine has been demonstrated to promote the absorption of intestinal polypeptides and amino acids, effectively improving the growth performance and meat quality traits of broilers [137,138,139]. Specifically, L-theanine supplementation in broilers’ diets has been found to enhance antioxidant enzymes’ activity and reduce oxidative damage, potentially related to the conversion of muscle fibers [140]. In cattle, L-theanine is used to mitigate the adverse effects of inflammation during periods of heat stress [141]. Current research on L-theanine has primarily focused on pigs and chickens, emphasizing enhanced growth performance and immune function.

Resveratrol, a natural antioxidant feed additive, promotes the conversion of Type II fast muscle fibers to Type I slow muscle fibers through the AMPK/SIRT1/PGC-1α signaling pathway [142,143,144]. Adding resveratrol to the diet of fattening pigs and sows increases MyHC-I and MyHC-IIA muscle fibers, decreases MyHC-IIB muscle fibers, and improves the muscles’ antioxidant metabolism, enhancing the taste and tenderness of pork [145,146]. Supplementing resveratrol in the diet of cattle effectively increases the proportion of Type I oxidized muscle fibers, improving the tenderness, color stability, stability, and antioxidant capacity of beef [147,148]. In Pekin ducks, adding 300–450 mg/kg of resveratrol induces the conversion of Type II glycolytic muscle fibers to Type I oxidized muscle fibers and reduces the cross-sectional area of Type II fibers, improving the tenderness and reducing the shear force of duck meat [149].

The addition of ractopamine to diets reduces the number of MyHC-IIA and MyHC-IIX muscle fibers while increasing MyHC-IIB muscle fibers, indicating a shift towards faster-twitch types of muscle fiber [150]. Studies have demonstrated that supplementing diets with 0.25% leucine promotes the expression of genes related to slow muscle, inhibits genes related to fast muscle, and induces the conversion of muscle fibers (Type II → Type I) [151]. Chen et al. found that adding 0.08% apple polyphenols to the diets of fattening pigs induced a transition from fast-twitch to slow-twitch muscle fibers, promoting mitochondrial biogenesis and enhancing the muscles’ antioxidant capacity [152]. Supplementing diets with dihydromyricetin in fattening pigs has been shown to facilitate the formation of slow muscle fibers and induce the conversion of fast to slow muscle fibers [153]. Bee observed that sows subjected to restricted feeding exhibited a reduction in the number of fast glycolytic (IIX, IIB) muscle fibers and an increase in the number of fast oxidative-glycolytic (IIA) muscle fibers at the same slaughtering age [154]. The proportion of Type I and Type IIA muscle fibers increased, while the proportion of Type IIX decreased, indicating a shift towards an oxidative type of muscle fiber with zinc methionine supplementation [155]. Supplementing lambs’ diets with α-lipoic acid has been shown to promote an increase in oxidized muscle fibers and a decrease in glycolytic muscle fibers, and to regulate the conversion of the type of muscle fibers (Type II → Type I) [156]. Spooner et al. fed Iberian pigs with high-fructose and high-fat diets, showing that Type I oxidized slow muscle fibers decreased and converted to Type II glycolytic fast muscle fibers, leading to an imbalance in the metabolism of skeletal muscles in vivo [157]. Similarly, a high-nutrient diet was found to increase Type IIB white muscle fibers and decrease the number of Type I and Type IIA red muscle fibers in broilers [78]. The addition of high-concentrate feed to a goat’s diet facilitated the conversion of intermediate muscle fibers to slow muscle fibers, thereby modifying the muscle fibers’ composition and enhancing the meat quality [158]. S J Meale et al. explored residual feed intake (RFI) in beef cattle and found a lower proportion of white muscle fibers in the gluteus medius muscle with H-RFI [159].

The morphological and spatial distribution of mitochondria in animal skeletal muscle is intimately linked to the specific type of muscle fiber, playing a crucial role in the occurrence of oxidized energy metabolism in muscle fibers and the biological process of muscle fibers’ transformation (Type II → Type I) [17,160,161]. Nutrients have been shown to enhance mitochondrial function and facilitate muscle fibers’ transformation. For instance, Chen et al. identified that administering a diet containing docosahexaenoic acid (DHA) to mice enhanced the expression of genes associated with adipose tissue and reorganized the m^6^A/DDIT4/PGC-1α signaling pathway, promoting mitochondrial biogenesis and the formation of slow muscle fibers [162]. Dietary supplementation with β-hydroxy-β-methylbutyrate (HMB) was observed to enhance the expression of the *PGC-1α* gene, thereby promoting mitochondrial biogenesis and the conversion to Type I oxidized muscle fibers [163].

Furthermore, maternal nutrition and health during pregnancy have a significant impact on the development of the muscle of offspring. Adequate and balanced nutrition supports the formation and differentiation of fetal muscle fibers, ensuring the potential for muscle growth after birth. Conversely, nutritional deficiencies or imbalances can lead to a reduced number of fetal muscle fibers and impaired growth potential. The mother’s health status, including chronic diseases, infections, and stress, also affects the development of fetal muscle. Chronic maternal diseases or complications of pregnancy can impact the development of fetal muscles through mechanisms such as insufficient placental blood and oxygen supply. Stress factors, such as excessive exercise and environmental pressure, can influence maternal hormone levels, thereby affecting the growth of fetal muscles.

Adding 25-hydroxycholecalciferol to the diet of pregnant sows has the potential to enhance the absorption of Vitamin D, which may increase the proportion of red meat in piglets while simultaneously reducing the fat content [164]. A high-fat diet during pregnancy can accelerate the growth of muscle mass in the offspring, leading to a proportional enlargement of the muscle fibers in the biceps femoris muscle and a shift in the muscle’s metabolic pattern towards glycolytic metabolism [165]. The expansion of primary muscle fibers and the upregulation of the gene *PPARα*, associated with Type I oxidized muscle fibers, in pregnant cows during grazing was more conducive to an increase in muscle mass in calves [166,167]. Conversely, restriction of maternal nutrients during pregnancy has been demonstrated to have a deleterious effect on the number and composition of muscle fibers in lambs [168,169]. However, the ratio of fast to slow muscles in lambs remains contentious, with potential influences including sex. During early pregnancy, maternal malnutrition will cause an imbalance in white and red muscles in the muscles of calves, resulting in a negative impact on meat quality [159]. Stickland also pointed out that maternal malnutrition in early pregnancy led to a decrease in the number of secondary myofibers muscle fibers in the offspring, but the primary muscle fibers were not affected [170]. Feeding sows on a high-fat/low-fiber diet resulted in decreased levels of MyHC-I protein, increased the levels of MyHC-IIX and MyHC-IIB protein, and impaired mitochondrial function in piglets [171]. Maternal linoleic acid supplementation in late pregnancy was beneficial for increasing the expression levels of MyHC-I mRNA and promoted the conversion of the type of muscle fibers to oxidative slow muscle [172]. Wang et al. found that a high-protein diet promoted the expression of MyHC-I, inhibited the expression of MyHC-II, and induced the formation of Type I muscle fibers in Meishan sows during pregnancy [173]. However, supplementing sows with extra nutrition during the second trimester of pregnancy (45–85 d) did not show any effect on the muscle development of their piglets [174]. In chickens, maternal supplementation with phytosterols esters [175], mulberry leaf flavonoids [176], and zinc [177] can improve the growth and development of skeletal muscles in the chicks. Restriction of maternal nutritional intake during pregnancy can lead to an increase in the diameter of muscle fibers and no change in the muscle mass in calves, thus negatively affecting the quality of carcass meat [178,179,180]. Fahey et al. found that the restriction of maternal nutrients promoted the formation of slow muscle fibers and inhibited fast muscle fibers in lambs before the formation of muscle fibers (30–70 d) [181]. Moreover, Ithurralde et al. confirmed that under conditions of extensive grazing, limited maternal nutrient would lead to an increase in the number of oxidized muscle fibers and a decrease in the number of glycolytic muscle fibers in lambs [168]. However, Nordby et al. showed that the restriction of maternal nutrients during early pregnancy did not affect the composition of muscle fibers in lambs [182].

#### 3.2.2. Environmental Temperatures

It is well established that livestock and poultry are homeothermic animals, meaning that an appropriate environmental temperature is beneficial to their health and productive performance. Domesticated animals of different ages and physiological stages require varying optimal environmental temperatures. Changes in the environmental temperature can lead to alterations in the composition of skeletal muscle fibers and the expression of related genes. Specifically, an optimal environmental temperature slightly below the required level can enhance cold tolerance, thereby increasing Type I oxidized muscle fibers.

For instance, the proportion of Type I oxidized muscle fibers increased in the semispinalis muscle of castrated male pigs raised at low temperatures, while the proportion of Type II muscle fibers decreased [183]. Low temperatures have been observed to induce the mRNA expression of MyHC-IIA and MyHC-I in piglets, while simultaneously decreasing the mRNA expression of MyHC-IIX, leading to conversion of the muscle fibers from Type IIA to Type I [184]. Sangwoo Kim et al. found that Mangalica pigs’ Type I muscle fibers increased in winter than in summer, and the conversion of the type of muscle fiber was regulated (Type II → Type I), thereby adapting to the cold environment [185]. Furthermore, early postnatal exposure to cold can significantly promote the upregulation of primary muscle fibers in piglets while inhibiting secondary fibers [186].

A short-term reduction in rearing temperatures resulted in the conversion of fast muscle fibers to slow muscle fibers in chicks. Furthermore, chicks with greater cold tolerance were found to have a higher proportion of slow muscle fibers [187,188]. Ferreira et al. found that the pectoral muscles of broilers reared at low temperatures exhibited greater redness, whereas those reared at higher temperatures had glycolytic muscle fibers with reduced cross-sectional areas, likely due to the greater abundance of capillaries in slow muscle fibers [189]. In cold environments, Type I muscle fibers increase, Type IIA muscle fibers decrease, and the muscles’ oxidative metabolism increases in Muscovy duck [190].

#### 3.2.3. Hormone Treatments

The skeletal muscles of animals are specialized tissues that maintain and regulate normal physiological and reproductive activities. The functionality and metabolic processes of these muscles largely depend on the characteristics and modes of action of their constituent muscle fibers [191]. Hormones, including growth hormones, β-agonists, insulin-like growth factors, and glucagon, play a crucial role in regulating the type and direction of the conversion of muscle fibers in domesticated animals [20].

Growth hormone (GH) is a peptide hormone that primarily affects skeletal muscle, promoting bone growth, muscle mass, and lipid metabolism [191,192]. Mjaaland et al. observed that the diameter of Type I muscle fibers in the soleus and gastrocnemius of piglets treated with GH increased, manifesting as hypertrophy of the muscle fibers [193]. In cattle, GH is used to facilitate protein synthesis in bovine skeletal muscle cells, while bovine somatotropin is used to induce cardiac hypertrophy in mice [194,195]. Hemmings et al. observed that GH had no impact on the mRNA expression of MyHC in the skeletal muscle of lambs, possibly due to the relatively short administration time (6 days).

β-agonists were found to stimulate an increase in the mRNA expression of MyHC-IIX and MyHC-IIB in lambs [196]. Carmelo Milioto et al. weakened the conversion from glycolytic to oxidative muscle fibers in mouse muscles by using β-agonists, in order to induce the hypertrophy of muscle fibers and improve motor function [197]. Additionally, β-adrenergic agonists do not appear to have an effect on the type of muscle fibers in lambs [198], but promote an increase in the muscle fibers’ cross-sectional area [199]. Ractopamine, as a β-adrenergic agonist, can promote an increase Type IIB muscle fibers in muscle sites, especially red muscles [200]. Gunawan et al. observed that as the duration of administering ractopamine increased, the expression of MyHC-IIA and MyHC-IIX gradually declined, while that of MyHC-IIB remained at a consistently elevated level [150]. Li et al. found a higher number of IIX–IIB hybrid muscle fibers in the longissimus dorsi muscle of castrated pigs treated with ractopamine [201]. In conclusion, Type IIB muscle fibers in the carcass muscles of pigs fed a diet containing rapamycin exhibited a general tendency to increase [202,203]. Oxidative muscle fibers (Type I and Type IIA) are more significantly affected by supplementation with clenbuterol in broilers’ diets and differ according to sex [204]. Daichi Ijiri et al. found an increase in the mass of the sartorius muscle after injection of the β2-adrenergic receptor agonist in chicks, but this failed to produce an effect on the pectoralis muscle [205]. Similarly, treatment of bovines’ longissimus dorsi muscle with beta-adrenergic agonists was found to promote the expression of *MLC-1f* (fast-twitch muscle fibers) [206]. Vestergaard et al. found that β-adrenergic agonists promoted an increase in Type IIB muscle fibers and a decrease in Type I and IIA muscle fibers, and reduced the oxidative capacity of the muscles in bulls [207].

Insulin-like growth factors (IGFs) are evolutionarily conserved peptides related to the structure of insulin [208]. Mature IGF-I and IGF-II are primarily composed of A, B, C, and D domains. IGF-1 is a crucial signal in anabolic and reparative processes within skeletal muscle tissue, orchestrating the proliferation and differentiation of myoblasts and influencing the regeneration and hypertrophy of muscle fibers [209,210,211]. In cases of liver fibrosis in chickens, muscle fibers in the pectoral muscle atrophy, while only Type II muscle fibers in the lateral part of the tibialis femoris muscle atrophy, with an increase in the number of Type I muscle fibers [122]. Nagasao et al. discovered that insulin-like growth factor 1 (IGF-1) was present in all muscle fibers of the lateral femoris tibialis muscle (Types I, IIA, and IIB), while IGF-1 receptor (IGF-1R) exhibited a robust positive correlation exclusively in Type I muscle fibers [212]. Consequently, IGF-1 can augment the number of Type I muscle fibers and stimulate the hypertrophy of muscle fibers via the IGF-1R pathway. However, the precise regulatory mechanisms remain to be fully delineated. Liu et al. observed an increase in muscle fibers’ diameter following the administration of IGF-1 to duck eggs, with a greater effect observed in the leg muscle (slow muscle fibers), suggesting a primary role for IGF-1 in the regulation of slow muscles [213]. Current research on IGF-1 in livestock has primarily considered its effects on the proliferation and differentiation of satellite cells of skeletal muscle and muscle mass, as well as the expression of IGF-1 during pregnancy [214,215,216,217,218]. Further studies are required to elucidate the regulatory mechanisms of the conversion of muscle fibers and muscle metabolism.

Glucagon, secreted by islet α-cells, indirectly influences the type of muscle fiber and the synthesis of skeletal muscle protein through its effects on the amino acid and insulin content [219,220]. Compared with foreign breeds, Chinese Tibetan pigs exhibit a greater abundance of Type I oxidized muscle fibers and a lower glucagon content in skeletal muscles, resulting in fresh meat with superior quality [221,222]. In Muscovy ducklings treated with glucagon, the muscle fibers present in the gastrocnemius muscle altered to oxidative slow muscle, and the oxidative metabolism of the muscle increased [190].

### 3.3. Genetic Regulation

Skeletal muscle is indispensable in livestock, playing a crucial role in their physiology. The growth and development of skeletal muscle involve a series of intricate biological processes. Initially, mesenchymal stem cells undergo terminal differentiation into myoblasts, which subsequently proliferate and differentiate into primary muscle fibers. These fibers mature into muscle fibers and undergo hypertrophy, eventually enhancing muscle mass. The biochemical and functional characteristics of skeletal muscle are influenced by the muscle fibers’ heterogeneity, which is intricately regulated by complex genetic mechanisms. Central to this regulation are non-coding RNAs and genes that collaborate in a sophisticated regulatory network. Over the past decade, research has focused extensively on understanding how these genetic factors, alongside signaling pathways, contribute to the regulation of muscle fibers’ heterogeneity.

In this review, we delved into the roles of non-coding RNAs, pertinent genes, and their interactions within these regulatory networks. By synthesizing recent findings, we aimed to provide a comprehensive overview of the genetic underpinnings that govern muscle fibers’ heterogeneity, shedding light on their implications for animal physiology and potential applications in various fields.

#### 3.3.1. Non-Coding RNAs

##### miRNA

Meat quality is crucial for the economic sustainability of livestock and poultry farming. Modern molecular breeding focuses on unraveling the molecular genetic mechanisms that regulate the development, growth, and transformation of skeletal muscle fibers. MicroRNAs (miRNAs) play pivotal roles in skeletal muscles’ growth, development, and metabolism as a class of gene transcription suppressors with conserved sequences. Approximately 22 nucleotides in length, miRNAs bind to mRNAs, leading to degradation of the target mRNA or the inhibition of protein translation [223], and are primarily involved in post-transcriptional regulation. Specifically, miRNAs highly expressed in skeletal muscle, known as myomiRs, include miR-1, miR-133, miR-208b, miR-206, miR-21, miR-499, and miR-128. They regulate the conversion of types of fiber and the dynamics of skeletal muscle mass in livestock and poultry [224,225].

For instance, the miR-1 SNP site in swine increases Type I and Type IIA muscle fibers, potentially influencing the composition of muscle fibers [226]. miR-152 is enriched in the slow muscle of pigs, with its expression being activated by *UCP3*, thus promoting the formation of slow muscle fibers [227]. miR-499-5p, expressed at higher levels in pigs’ slow muscles, promotes the formation of oxidative muscle fibers by inhibiting the *Sox6* gene [228,229]. Zhang et al. demonstrated that miR-133a-3p targeting *TEAD1* facilitated the formation of slow muscle fibers in pigs, while miR-208b and miR-499-5p targeting *Sp3* and *Sox6* similarly promoted the formation of slow muscle fibers [230]. miR-30 targets *Prdm1* to regulate the formation of fast-twitch muscles and the differentiation of muscle fibers in pigs [231]. miR-24-3p, highly expressed in the fast muscles of pigs, promotes the conversion of muscle fibers from Type I to Type II [232]. Its regulatory effects on genes that are highly expressed in slow muscle, including *Nek4*, *Pim1*, *Nlk*, *Pskh1*, and *Mapk14*, require further exploration. *MEF2C*, *NRF-1*, *mtTFA*, *Cytc*, and *SDH* are downstream targets of *PGC-1α*, which is associated with mitochondrial biogenesis and oxidative metabolism in muscles. miR-27a negatively regulates *PGC-1α*, thereby inhibiting its downstream targets and the mRNA expression of *MYH7*, which inhibits the formation of slow muscle fibers [233]. Conversely, Zhang et al. observed that overexpression of miR-27a increases the levels of MyHC-I protein and decreases the levels of MyHC-II protein, promoting transformation from fast to slow types of muscle fiber in pigs [234]. The hypothesis that porcine *FOXO1* is a target gene of miR-27a in the transformation of muscle fiber needs verification. Zuo et al. identified four differentially expressed miRNAs (DE-miRNAs) during the growth and development of muscles in pigs (at Days 63, 98, and 161), including ssc-miR-1, ssc-miR-143-3p, ssc-miR-92a, and ssc-miR-127, which are related to muscle fibers. Notably, ssc-miR-143-3p promotes the formation of slow muscle fibers by increasing *MYH7* protein via the HDAC4-MEF2 signaling pathway [235]. Ma et al. conducted deep sequencing of miRNA transcriptomes from 15 porcine skeletal muscles, identifying 18 DE-miRNAs involved in the generation of muscle, including miR-125b, miR-126, miR-128, miR-486, and miR-99a/b, directly influencing oxidative slow muscles via the IGF signaling pathway [236]. Furthermore, overexpression of miR-22-3p inhibits the expression of slow muscle fibers in pigs [237].

Zuo et al. demonstrated that miR-1 and miR-143 increase the number of slow muscle fibers via the HDAC4/MEF2 signaling pathway [235]. Liu et al., constructing a regulatory interaction network between miRNAs and mRNA related to the composition of muscle fibers in chickens, identified gga-miR-143-5p, gga-miR-499-5p, and gga-miR-129-3p as regulators of the transformation of the muscle fibers’ phenotype through the CaN/NFAT signaling pathway [238]. Furthermore, gga-miR-196-5p promotes the formation of slow muscle fibers in chicken by targeting *CALM1*. miR-499-5p is expressed in slow muscle fibers in chicken, promoting the formation of oxidized slow muscle fibers via targeting inhibition of the expression of *SOX6* [239]. Furthermore, *SOX6*, interacting directly with *RUNX2*, *PRDM1*, *HDAC4*, and *MYH7B*, is inhibited by miR-499-5p, which is expressed in slow muscle fibers in chicken and promotes the formation of oxidized muscle fibers. miR-1611, exclusively expressed in slow muscle fibers in chicken, facilitates the conversion from fast to slow muscle fibers by regulating *Six1* through lncRNA-Six1 [240].

Akihiko Horikawa et al. analyzed miRNA and mRNA in the skeletal muscle of grazing cattle and found a higher proportion of slow muscle fibers compared with caged-raised cattle. They highlighted miR-208, miR-206, and miR-10b as regulators of the types of muscle fiber, suggesting further investigations into their molecular mechanisms [241]. In Japanese Black cattle, Murasawa et al. identified specific miRNA expression patterns in fast and slow muscle types: the expression of miR-196a and miR-885 in fast muscle, and high miR-208b in slow muscle [242]. miR-196a potentially promotes the formation of bovine fast muscle fibers by targeting *HMGAI*, *SEMA3A*, *RICTOR*, *IGFI*, *IGF2BP1*, and *IGF2BP3*, while miR-208b synergistically promotes slow muscle fibers with MyHC-I. M. Greene et al. conducted a transcriptomic analysis during hypertrophy of skeletal muscle in lambs, identifying 115 differentially expressed miRNAs during the transition period from 133 days of gestation to 42 days after birth [243]. However, the precise roles of these miRNAs in growth, development, and differentiation of the type of fiber of skeletal muscles—including miR-22-3p, let-7g, miR-30c, miR-30d, miR-29a, miR-143, miR-299-5p, miR-487b-3p, miR-127, and miR-432—require further elucidation.

Overall, miRNAs play a crucial role in regulating the types of muscle fibers and their conversion, influencing the expression of MyHC subtype proteins and indirectly impacting meat quality through their effects on the composition of muscle fibers (Table 2).

##### LncRNA

Long non-coding RNA (lncRNA) refers to RNA molecules longer than 200 nucleotides that play crucial roles in regulating transcription and post-transcriptional processes involved in the growth and development of skeletal muscle [244]. Wang et al. conducted MeRIP-seq analysis of M^6^A-modified lncRNAs and identified seven differentially methylated lncRNAs (DME-lncRNAs) in porcine skeletal muscle (MSTRG.14200.1, MSTRG.2082.1, MSTRG.19265.9, MSTRG.17296.1, MSTRG.13515.1, MSTRG.2121.6, and ENSSSCT00000074465) that may influence the conversion of muscle fibers through the regulation of cis-target genes [245]. Notably, knockdown of MSTRG.14200.1 has been shown to facilitate the transition from fast to slow muscle fibers. Dou et al. identified MyHC-IIA/X-AS as a lncRNA in pigs, finding that spongy miR-130b can promote the expression of MyHC-IIX, inhibit the expression of MyHC-I, and increase the number of fast types of muscle fibers [246]. Shen et al. analyzed Qingyu pigs and identified lncRNAs involved in regulating the phenotypes of muscle fiber through cis-regulation. They highlighted two circRNA–miRNA–mRNA axes (circRNA290-miR-27b-Foxj3 and circRNA9210-miR-23a-MEF2C) related to the conversion of fast and slow muscle fibers [247]. Wang et al. constructed ceRNA expression profiles of the longissimus dorsi muscle in Huainan pigs and DLY, identifying differentially expressed mRNAs, lncRNAs, and circRNAs associated with the conversion of muscle fiber, regeneration of skeletal muscle, and differentiation [248].

In chickens, lncRNAs such as lncRNA-Six1, lncRNA-FKBP1C, LCC-EDCH1, and LCC-IRS regulate muscle development and fiber types through mechanisms including the activity of miRNA sponges and encoding short micropeptides [249]. For instance, lncRNA-FKBP1C interacts with *MYH1B* to stabilize its protein, promoting the conversion from fast to slow muscle fibers [250]. Ju et al. analyzed Chinese Qingyuan partridge chickens and constructed lncRNA-miRNA regulatory networks, demonstrating how lncRNAs influence the types of muscle fibers through interactions with the target genes [121]. Notably, lncRNAs such as lncXR_001466942.2, lncXR_003077811.1, lncXR_001465741.2, and lncXR_003074785.1 are associated with oxidized types of muscle fibers.

Analyzing yaks’ muscle tissue, Wu et al. identified mRNA, lncRNA, and circRNA involved in regulatory networks affecting the conversion of the type of muscle fibers, highlighting lnc-005603-PPARGC1A among the identified lncRNAs potentially involved in determining the type of muscle fiber in yaks [126]. Tugeqin Bou et al. studied Mongolian horses and found specific miRNA binding sites on highly expressed lncRNAs in the gluteal medium muscle, influencing the regulation of the type of muscle fibers [129]. These studies collectively underscore the diverse roles of lncRNAs in regulating the types of muscle fibers and their potential applications in livestock breeding and improvements in meat quality.

##### CircRNA

Circular RNA (circRNA) is an endogenous, covalently closed loop non-coding RNA that functions by sponging miRNAs and proteins, and by regulating host genes’ transcription and translation [251]. Studies have highlighted circRNAs’ role in regulating genes associated with types of muscle fibers in livestock and poultry. Cao et al. identified 181 differentially expressed (DE-) circRNAs in the porcine longest dorsal muscle and flounder muscle, constructing a circRNA–miRNA interaction network. Among these, circMYLK4 has been shown to promote the formation of porcine slow muscle fibers and myofibers by upregulating mitochondrial biogenesis and myofiber-specific genes’ expression [252]. However, the detailed molecular mechanisms underlying these processes require further investigation. Zhuang et al. investigated circRNA–miRNA–mRNA networks in Lantang pigs of different ages, revealing that overexpression of circKANSL1L promotes the expression of MyHC-I and MyHC-IIB, influencing differentiation of the types of muscle fiber [75].

Yang et al. constructed a competing endogenous RNA (ceRNA) regulatory network in bovine longissimus dorsi muscle associated with circRNAs, finding that circRNAs affect the expression of β-MHC and α-MHC in MyHC-I by competitively splicing *MYH6* and *MYH7*, impacting the metabolism of oxidized muscle fiber [253]. In yaks, novel_circ_0006099 sponges miR-129 to regulate *MYH7*, influencing the determination of the type of slow muscle fiber [126]. Overexpression of circUSP13 in goats regulates mitochondrial homeostasis and autophagy via the IGF1/MAPK/ERK pathway, promoting the upregulation of MyHC-slow and the downregulation of MyHC-fast, thus converting muscle fibers from fast to slow [254].

These studies collectively emphasize the involvement of lncRNAs and circRNAs in the development and conversion of myofiber in livestock and poultry. Despite advancements, only a few non-coding RNAs have been confirmed to influence the transformation of the type of muscle fiber (Table 3). Current research in livestock has primarily focused on adipocytes, the myocardium, and constructing regulatory networks involving non-coding RNAs. Future efforts should prioritize identifying key lncRNAs and circRNAs crucial related to muscles’ growth and development, and determination of the type of fiber in livestock and poultry.

#### 3.3.2. Marker Genes of Myofiber Transformation 

##### The MYHs Family

*MYH*, a crucial component of myosin, plays a pivotal role in determining the differentiation and specialization of various types of muscle cell, thereby influencing the growth and development of muscle and bone in animals [255,256]. Among the *MYH* gene family, *MYH3* is particularly significant in overseeing the growth and development of skeletal muscle during embryogenesis, as well as in the regeneration and differentiation of muscle fibers in adulthood [257]. In comparison with commercial pig breeds such as Landrace, Korean native black pigs exhibit higher levels of red coloration and marbling, attributes associated with the *MYH3Q* variant that promotes the formation of Type I oxidized slow muscle fibers [258]. Brown et al. discovered a 3 bp difference in the CArG/E-box region of the *MYH4* promoter between humans and pigs, which enhances myogenesis in pigs and facilitates the formation of glycolytic muscle fibers [259]. Eun-Seok Cho et al. found a novel SNP (g.-1398G>T) in the *MYH4* gene of Landrace pigs, correlating it with the number and cross-sectional area of Type IIA myofibrils, though further research is needed to understand its post-transcriptional regulatory mechanisms [260]. However, further studies are required in order to elucidate the underlying mechanisms by which the MYH4 mutation site regulates the distribution of myofibrils at the post-transcriptional level.

*MYH14*, analogous to slow myosin 2 in chicken, encodes mammalian slow tonic myosin and plays a role in slow twitch muscle fibers in chicken [261]. *UCP1*, in conjunction with *MYH* family genes, influences the formation and development of fast-twitch muscle fibers in beef cattle, as demonstrated by Diao et al. [262]. In fish muscle, the regulation of the *MYH* family is crucial for differentiating various types of muscle fiber. For instance, *MYH_M86-2_* is specifically expressed in the slow muscle fibers of zebrafish [263]. Chen et al. observed the differential expression of *MYH* genes among the types of muscle fiber in Chinese perch, where *MYH7aa*, *MYH13*.3, *MYHb.1*, and *MYH9b* predominated in red muscle, while *MYHs* was predominantly expressed in white muscle, constituting over 90% of the total expression of the *MYH* family [264].

##### PGC-1α

Peroxisome proliferator-activated receptor coactivator-1 (*PGC-1*) is pivotal in enhancing mitochondrial biogenesis and oxidative metabolism in muscle, playing a crucial role in the metabolism of skeletal muscle [265]. Within the *PGC-1* family, *PGC-1α* stands out as the foremost member, recognized for inducing mitochondrial biosynthesis in skeletal muscle and promoting the transformation of the type of muscle fiber [266].

Ying et al. demonstrated, through a transgenic pig model, that *PGC-1α* stimulates the growth of oxidized muscle fibers, reduces glycolytic muscle fibers, and induces transformation of the type of fiber (Type II → Type I) by upregulating *MyOG* and downregulating *MSTN* and *FOXO1* [91]. Zhang et al. further found that *PGC-1α* facilitates the conversion of glycolytic Type II fibers to oxidative Type I and Type IIA fibers, enhancing meat’s redness through increased mitochondrial oxidative respiration and fatty acid metabolism [68]. *PGC-1α* also suppresses the expression of *MYH7* by negatively regulating the factors involved in mitochondrial biogenesis and oxidative metabolism, thereby inhibiting the production of slow muscle fibers in pigs [233]. Supplementation of butyrate in pigs’ diets increases *PGC-1α* mRNA and protein levels, promoting the expression of mitochondrial genes (*NRF1*, *TFAM*, *Cytc*) and the formation of slow muscle fiber [230]. Gu et al., using *PGC-1α*-overexpressing pigs, identified 184 upregulated and 187 downregulated genes affecting the conversion of muscle fibers through pathways related to fat metabolism [267]. Elevated expression of *PGC-1α* activates PPARδ signaling, upregulating the expression of miR-208 and miR-499, thereby increasing Type I muscle fibers [268].

In chickens, *PGC-1α* and its haplotype polymorphisms influence the type of muscle fiber [269]. Cold exposure in chicks upregulates *PGC-1α* and downregulates *MSTN*, shifting the differentiation of muscle fibers from fast to slow [270]. Shu et al. studied Qingyuan pheasants, linking *PGC-1α* gene polymorphisms (G646A) and haplotypes (H1) to conversion of the type of muscle fiber type and meat quality [271].

Khan et al. observed the niacin-induced transformation of sheep muscle fibers from Type II to Type I, accompanied by increased *PPARGC1A* (encoding *PGC-1α*) mRNA levels [272]. However, the direct involvement of *PGC-1α* in the composition and conversion of muscle fibers warrants further investigation. While bovine *PGC-1α* studies have primarily focused on adipose tissue and mitochondrial biogenesis, its role in skeletal muscle suggests its potential involvement in the formation and transformation of fast and slow fibers [273,274,275]. Table 4 summarizes the relevant studies on the influence of the *MYHs* family and *PGC-1α* on the regulation of muscle fibers’ heterogeneity.

##### Other Genes

The *MSTN* gene modulates ratios of muscle fibers by regulating *MRF*, *MEF2C* [276], *MyOD* [277], and *MyOG* [278], increasing Type II and decreasing Type I fibers in MSTN-knockout pigs. Li et al. used CRISPR/Cas9 to edit the *MSTN* signal peptide, preserving the expression of mature *MSTN* peptides, promoting muscle growth in pigs, impacting oocyte quality, glucose tolerance, and metabolism [279]. Gu et al. found that increased CAMKII and phosphorylation of p38 MAPK in *PPARγ* pigs promotes the formation of oxidative slow muscle fibers via *MEF2* and *PGC-1αZ* [280]. *FHL3* affects the binding of YY1 to MyHC-IIB regulatory regions, increasing the number and size of fast muscle fibers in pigs [281]. *Prox1* in porcine muscle correlates positively with MyHC-I and inversely with MyHC-IIB, enhancing the conversion of skeletal muscle fibers and the quality of fresh meat [282]. It can enhance the quality of fresh meat by regulating the conversion of muscle fibers in skeletal muscle, although the specific mechanism of action remains to be elucidated. In addition, Wei et al. used iTRAQ proteomics to screen for differential proteins (DAPs) in fast and slow muscles of pigs, and found that the DEGs and DAPs of *MYBPC1*, *MYH7*, *ACTN2*, *ANKRD2*, *MYL3*, *TNNC1*, *LMCD1*, *CSRP3*, *TNNT1*, *TNNI1*, *HSPB6*, and *ENSSSCG0000039506* (novel genes) overlapped and were key candidate genes for the formation of muscle fibers [283].

In chicken muscle, *SOX6* activates *MEF2C*, promoting the development of slow-twitch fibers while inhibiting *Nfix*. Overexpression of *Nfix* counteracts *SOX6*’s influence on *MEF2C* [284]. *ANKRD2*, conserved in mammals and poultry, preferentially expresses in red slow muscle fibers of chicken (Types I and IIA), impacting the conversion of muscle fibers [285]. Wang et al. identified DEGs such as *FHL1*, *MYBPC1*, and *MYH3* in chickens’ skeletal muscles, implicating glycolysis, insulin signaling, and proliferation of myoblasts in the conversion of the type of fiber [286]. These differentially expressed genes may be involved in the proliferation of smyoblast and the conversion of slow muscle fibers into fast muscle fibers. *Myoz3* in Yellow Bantam chickens promotes the expression of the fast muscle fiber marker *MYH1F* via PPAR signaling, regulating the type of muscle fiber [287,288]. Weimer et al. identified an E47-interacting E-box in the slow MyHC-IIA promoter that was crucial for regulation of the muscle promoter’s activity [289]. The high expression of *CAST* in myocardial and skeletal muscle of chicken suggests a role in the development of muscle fibers [290]. BTEB1/KLF9 binds the *SP1* promoter’s cis-element to activate *FGFR1*, regulating myogenesis and muscle differentiation in poultry [291].

In double-muscled Belgian Blue cattle, deletion of *MSTN* increases Type IIB and decreases Type I and IIA fibers by upregulating *MyoD* and downregulating *MEF2C* [292]. *F94L* enhances Type IIA and IIX muscle fibers in cattle [293]. *ADAM12* in bovine slow muscle fibers co-localizes with Type I fibers, influencing the types of muscle fiber via phosphorylation and glycosylation [294]. In particular, the *ACTN3* gene is expressed in Type II muscle fibers of cattle [295]. Soria et al. found that the distribution of bovine muscle fibers was independent of *PPARGC1A* SNP 1181 [296]. β-agonists in lambs upregulate *RIP140* and downregulate *PGC-1β* via the PPARδ pathway, promoting the formation of fast muscle fiber, while *Six1* and *Eya1* are downregulated [196]. Yu et al. studied the molecular mechanism affecting muscle hypertrophy in Callipyge sheep, and pointed out that *RTL1* and *DLK1* synergistically induce muscle hypertrophy, and that *DLK1* dominates and directly targets the upregulation of *DNTTIP1* and *PDE4*, thereby promoting the upregulation of *MYH4*, the downregulation of *MYH7*, and the expression of fast muscle fibers [297].

Table 5 summarizes the specific molecular mechanisms involved in regulating the composition and transformation of muscle fibers in livestock. Genes exert their influence through various mechanisms such as mutation of promoter sites, overexpression, inhibition, and targeted regulation of non-coding RNAs. Numerous studies have identified candidate genes that exhibit differential expression in the fast and slow muscles of livestock. However, there remains a significant gap in verifying the precise relationships of the upstream and downstream targets of transcription factor associated with these genes.

##### Signaling Pathways

During the growth and development of animal bodies, external stimuli alter the concentration of Ca^2+^ and metabolite levels in skeletal muscle cells, subsequently activating downstream signaling pathways. This process regulates the expression of muscle fiber-specific genes, inducing adaptive transformations in the muscle fibers. In the longissimus dorsi muscle of pigs, increased expression of Akirin2 promotes mRNA levels of MyHC-IIB while reducing MyHC-I and MyHC-IIA mRNA. Simultaneously, key genes in the calcineurin/NFATC1 pathway (*NFATc1*, *PGC-1α*, *MEF2C*, *MCIP1*) are suppressed, shifting the types of muscle fibers from slow to fast [298]. In weaned piglets, ferulic acid enhances the expression of mitochondrial transcription genes (*COI*, *NRF1*, *mtTFA*, *mtTFB*, *cytc*) through the Sirt1/AMPK/PGC-1α pathway, promoting the formation of slow muscle fibers and improving the quality of pork [299]. *FNDC5* positively regulates MyHC-IIA mRNA via the PGC-1α/FNDC5 axis, which is crucial for controlling porcine intermediate muscle fibers [300]. *FOXO1* facilitates the growth of fast muscle fibers while inhibiting the development of slower phenotypes through the PI3K/PKB, MAPK/ERK, and Calcineurin/NFAT pathways [301]. Leucine promotes the transformation of porcine muscle fibers from fast to slow by activating the Akt/FOXO1 pathway [234]. In particular, fatty acid metabolism, degradation, and elongation pathways may be involved in the formation of the types of skeletal muscle fiber [283].

In poultry, IGF-1 influences protein synthesis in glycolytic pectoral muscles via the AKT/S6 pathway [302]. The coordination between IGF-1 and *MSTN* affects gene expression, whereas protein synthesis in oxidized adductor superficialis muscles appears to be independent of the AKT pathway [303]. Du et al. identified 1318 DEGs and 18 metabolic pathways related to the types of chicken muscle fiber, and implicating Hedgehog and Calcium signaling as a key for regulating the composition of muscle fibers [304].

Resveratrol activates the AMPK/SIRT1/PGC-1α pathway, promoting slow types of muscle fiber (increased MyHC-IIA and MyHC-I proteins; decreased MyHC-IIB and MyHC-IIX proteins) in cattle [274]. Pueraria regulates AMPK/PGC-1α/Nrf1 signaling, reducing Type IIB muscle cells and enhancing antioxidant capacity in bovine muscle [305]. All-trans retinoic acid (ATRA) via PPARδ signaling induces the formation of oxidized muscle fibers and inhibits the formation of glycolytic muscle fibers in cattle [306]. Vitamin A in sheep activates the p38 MAPK/PGC-1α pathway, increasing MyHC-I fibers and decreasing MyHC-IIX fibers, thereby stimulating mitochondrial biogenesis [307]. Herding sheep promote the conversion of Type II to Type I muscle fibers through the AMPK/PGC-1α pathway [308]. The *AMPKα2* and *PGC-1α* genes have been identified as key regulators of the type of muscle fiber and meat quality in Mongolian sheep. *DLK1* targets *PARK7* and *PDE4D*, promoting the expression of *MYH4* via the cAMP pathway and inhibiting *MYH7* via the PI3K/AKT pathway, which enhances muscle hypertrophy and the formation of fast fibers in Callipyge sheep. [297]. In yaks, differential mRNA expression of *MYH7*, *TNNT1*, *TPM3*, and *MYL3* influences the distribution of muscle fibers via the PPAR and PI3K/Akt signaling pathways [126].

**Figure 2:**
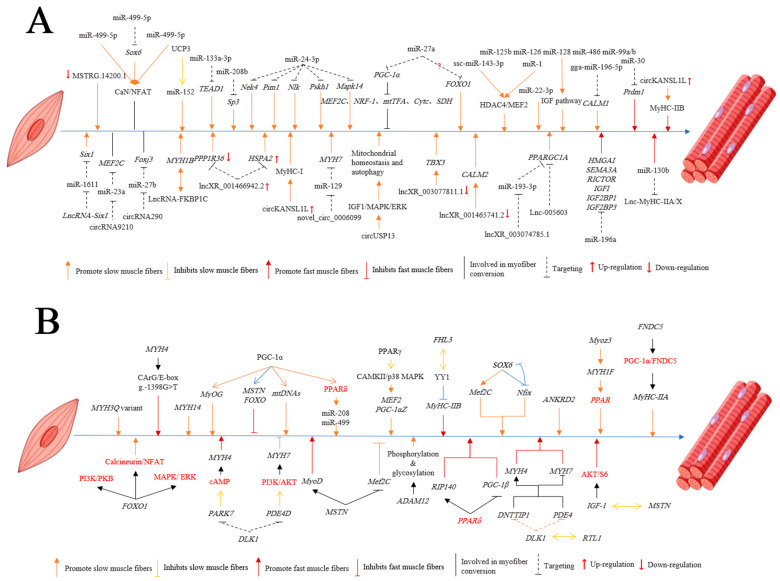

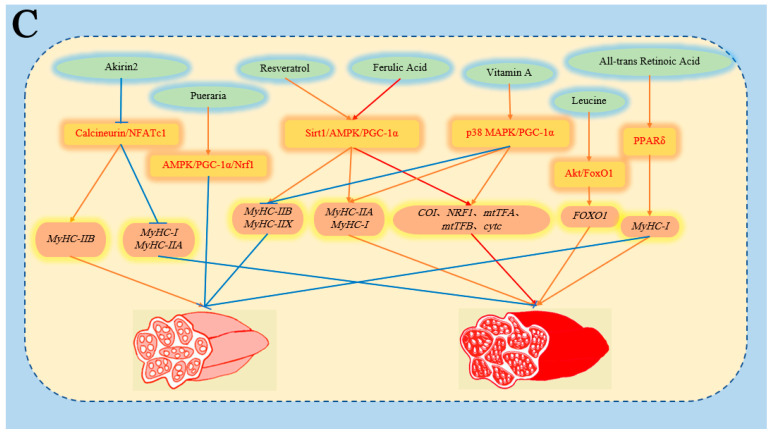
Regulation of the types of muscle fibers by non-coding RNAs, genes, and signaling pathways. (**A**) Regulation of the types of muscle fibers by miRNAs, lncRNAs, and circRNAs. (**B**) Regulation of the types of muscle fiber by genes and signaling pathways. (**C**) Other substances regulating the type of muscle fibers through signaling pathways.

Table 6 provides a summary of the regulatory factors influencing the composition of muscle fibers in livestock through various signaling pathways. These pathways include PI3K/AKT, PPAR, MAPK, and AMPK, primarily regulated by non-coding RNAs and genes. MiRNAs and genes have been extensively studied in regarding control of the type of muscle fiber, while lncRNAs and circRNAs mainly form regulatory networks, although their target relationships are less explored (Figure 2). Current research has focused on understanding how non-coding RNAs and genes orchestrate the differentiation of muscle fibers. Notably, there is a lack of studies investigating the mechanisms behind the conversion of fast to slow muscle fibers.

## 4. Conclusions and Prospects

As societal demands for high-quality meat products increase globally, understanding the factors influencing myofibers’ heterogeneity in livestock has become crucial. The composition of myofibers significantly impacts the appearance, flavor, and texture of meat. This heterogeneity is influenced by factors at various biological levels, including genetic regulation, diet, age, sex, environmental temperature, and muscle area.

Factors such as breed, age, and environmental temperature have notable effects on the types of muscle fibers in livestock. Crossbreeding to optimize the composition of muscle fibers incurs costs in terms of time, resources, and potential stress on animals. While the impact of sex on muscle fibers’ heterogeneity has been observed in some specific cases, such as rabbit masseter muscles, its significance remains uncertain in other species such as pigs, chickens, cattle, and sheep. The deep muscles are primarily composed of oxidized muscle fibers, while the shallow muscles are composed of glycolytic muscle fibers. Nutrition regulation and hormonal differences directly influence the types of muscle fibers, though the specific mechanisms have not been fully elucidated.

Advances in sequencing and omics technologies have identified numerous non-coding RNAs (ncRNAs) and genes involved in the conversion of muscle fibers, often interacting with key signaling pathways such as calcineurin/NFATc1, PI3K/Akt, MAPK, AMPK, cAMP, and PPAR. MiRNAs, in particular, play a significant role by targeting the downstream genes involved in fast and slow phenotypes of muscle fibers. In contrast, lncRNAs and circRNAs mainly regulate the distribution and transformation of muscle fibers through ceRNA networks, though their main effects and functional roles are still being explored. Genes directly or indirectly regulate the formation and conversion of muscle fibers through various signaling pathways, influencing genes related to the *MYH* family (MyHC-IIB, MyHC-I, MyHC-IIA, and MyHC-IIX), mitochondrial biogenesis, and oxidative metabolism (*MSTN*, *PRKAG1*, *PRKAG3*, and *PRKAR2B*). Despite the identification of numerous candidate ncRNAs and genes, validating their specific regulatory roles remains a challenge.

Future research should emphasize molecular genetic regulation’s impact on myofibers’ heterogeneity in livestock, integrating modern histological techniques to construct genetic regulatory networks and verify the regulatory mechanisms at the single-cell level. Respecting natural processes of growth and development will be essential in optimizing the composition of muscle fibers to meet dietary needs sustainably. Ultimately, establishing a comprehensive framework of the factors influencing muscle fibers’ heterogeneity will advance measurement standards of meat quality and facilitate the production of superior meat products to meet evolving consumer preferences worldwide.

## Figures and Tables

**Table 1:** Characteristics of different muscle fibers.

Type of Muscle Fiber and Traits	Type I	Type IIA	Type IIX and Type IIB
Color	Bright red	Red	White
Twitching speed	+	++	+++
Characteristics of energy metabolism	Oxidative metabolism	Oxidative metabolism and glycolytic metabolism	Glycolytic metabolism
Fatigue resistance	+++	++	+
Cross-sectional area (CSA)	+	++	+++
Glycogen	+	++	+++
Mitochondrial content	+++	++	+
ATPase activity	+	++	+++
Exercise	Low-intensity endurance	Moderate-intensity aerobic	Short bursts of high intensity

“+” positive regulation.

**Table 2:** Regulation of miRNAs on the type and transformation of muscle fibers.

Species	miRNAs	Target Genes	Regulation of the Types of Muscle Fiber			References
			Slow Muscle	Fast Muscle	Transformation of Muscle Fibers	
*Sus scrofa*	miR-1	/	+	/	/	[226]
	miR-152	/	+	/	/	[227]
	miR-499-5p	*SOX6*	+	/	/	[228,229]
	miR-30	*Prdm1*	/	+	/	[231]
	miR-24-3p	*Nek4*, *Pim1*, *Nlk* *Pskh1*, *Mapk14*	−	+	Slow muscle → fast muscle	[232]
	miR-27a	*PGC-1α*	−	+	/	[233]
		*FOXO1*	+	−	Fast muscle → slow muscle	[234]
	miR-133a-3p	*TEAD1*	+	−	/	[230]
	miR-208b miR-499-5p	*Sp3* *Sox6*	+	−	/	
	ssc-miR-143-3p	*MYH7*	+	/	/	[235]
	miR-125b miR-126 miR-128 miR-486 miR-99a/b	*IGF-1*	+	−	/	[236]
	miR-22-3p	*/*	−	+	/	[237]
*Gallus gallus*	gga-miR-143-5p gga-miR-499-5p gga-miR-129-3p	*CaN/NFAT* signalling pathway	+	/	/	[238]
	gga-miR-196-5p	*CALM1*	+	/	Fast muscle → slow muscle	
	miR-499-5p	*SOX6*	+	−	Fast muscle → slow muscle	[239]
	miR-1611	*Six1*	+	/	Fast muscle → slow muscle	[240]
*Bos taurus*	miR-208 miR-206 miR-10b	*/*	/	/	+*	[241]
	miR-196a miR-885	*HMGAI*, *SEMA3A* *RICTOR*, *IGFI* *IGF2BP1*, *IGF2BP3*	−	+	/	[242]
	miR-208b	*/*	+	−	/	

“+” positive regulation; “−” negative regulation; “/” no regulation; “+*” The regulation of muscle fiber type is related to this process, but the direction of regulation has not yet been determined.

**Table 3:** Regulation of the types and transition of myofiber by lncRNAs and circRNAs.

Species	lncRNAs/circRNAs	Possible Mechanism of Action	Regulation of the Types of Muscle Fibers			Reference
			Slow Muscle	Fast Muscle	Transformation of Muscle Fibers	
*Sus scrofa*	MSTRG.14200.1	After knockdown, the expression of MyHC-I protein is upregulated and the expression of MyHC-IIB protein is downregulated	+	−	Fast muscle → slow muscle	[213]
	Lnc MyHC--IIA/X	Sponging miR-130b	−	+	Slow muscle → fast muscle	[246]
	circRNA290	Sponging miR-27b, targeting *Foxj3*	/	/	+	[247]
	circRNA9210	Sponging miR-23a, targeting *MEF2C*	/	/	+	[247]
	circMYLK4	Regulates mitochondria-related factors	+	/	/	[252]
	circKANSL1L	After overexpression, the expression of MyHC-I and MyHC-IIB is upregulated	+	+	+*	[75]
*Gallus gallus*	LncRNA-Six1 LncRNA-FKBP1C Lnc-EDCH1 Lnc-IRS1	Cis-regulator or trans-regulator Sponging miRNAs Coding short molecular micropeptides	/	/	+* (Oxidation type)	[249]
	LncRNA-FKBP1C	Specifically interacts with *MYH1B* and enhances the stability of *MYH1B* protein	+	−	Fast muscle → slow muscle	
	lncXR_001466942.2	After overexpression, *PPP1R36* was downregulated and *HSPA2* was upregulated	+	/	/	[121]
	lncXR_003077811.1	After knockdown, *TBX3* was targeted and upregulated	+	/	/	
	lncXR_001465741.2	After knockdown, *CALM2* was targeted and downregulated	+	/	/	
	lncXR_003074785.1	Sponging miR-193-3p, targeting *PPARGC1A*	+	/	/	
*Bos taurus*	ceRNA	Competitive linear splicing of *MYH6* and *MYH7*	−	/	/	[253]
*Capra hircus*	circUSP13	After overexpression, mitochondrial homeostasis and autophagy are regulated through the IGF1/MAPK/ERK signaling pathway	+	−	Fast muscle → slow muscle	[222]
*Bos mutus*	Lnc-005603	Targeting *PPARGC1A*	/	/	+*	[126]
	novel_circ_0006099	Sponging miR-129, targeting *MYH7*	+	/	+*	

“+” positive regulation; “−” negative regulation; “/” no regulation; “+*” The regulation of muscle fiber type is related to this process, but the direction of regulation has not yet been determined.

**Table 4:** Regulation of the type and transition of myofibers by the *MYH*s family and *PGC-1α*.

Species	*MYHs* Family/*PGC-1α*	Possible Mechanism of Action	Regulation of the Type of Muscle Fibers			Reference
			Slow Muscle	Fast Muscle	Transformation of Muscle Fibers	
*Sus scrofa*	*MYH3*	Through the *MYH3Q* variant	+	/	/	[258]
	*MYH4*	A 3 bp difference in the CArG/E-box area	−	+	Slow muscle → fast muscle	[259]
		The SNP g.-1398G>T site regulates the number and cross-sectional area of Type IIA muscle fibers	+	/	/	[260]
	*PGC-1α*	Negatively regulates the expression of mitochondrial genes and inhibits oxidative metabolism	−	+	Slow muscle → fast muscle	[233]
		Positively regulates the expression of mitochondrial genes and promotes oxidative metabolism	+	−	Fast muscle → slow muscle	[230]
		Upregulates *MyOG*, and downregulates *MSTN* and *FOXO1*	+	−	Fast muscle → slow muscle	[91]
		Enhances the function of mitochondrial oxidative respiration and fatty acid oxidative metabolism	+	−	Fast muscle → slow muscle	[68]
		Through the fat metabolic pathway	/	/	+*	[267]
		Activates the PPARδ signaling pathway, and upregulates the expression levels of miR-208 and miR-499	+	/	/	[268]
*Gallus gallus*	*MYH14*	Codesslow tonic myosin	+	/	/	[261]
	*PGC-1α*	Upregulated expression of *PGC-1α*	+	−	Fast muscle → slow muscle	[270]
		G646A gene and H1 haplotype	/	/	+*	[271]
*Bos taurus*	*MYHs family*	Interacts with UCP1	/	+	/	[262]
*Capra hircus*	*PGC-1α*	After administration of niacin, levels of *PPARGC1A* mRNA, which encodes *PGC-1α*, increased	+	−	Fast muscle → slow muscle	[272]
*Danio rerio*	*MYH_M86-2_*	*Sox6* acts as a suppressor of transcription	+	−	/	[263]

“+” positive regulation; “−” negative regulation; “/” no regulation; “+*” The regulation of muscle fiber type is related to this process, but the direction of regulation has not yet been determined.

**Table 5:** Other genes that regulate the type and transformation of muscle fibers.

Species	Genes	Possible Mechanism of Action	Regulation of the Types of Muscle Fiber			Reference
			Slow Muscle	Fast Muscle	Transformation of Muscle Fiber	
*Sus scrofa*	*MSTN*	After knockout, the expression of *MRF*, *MEF2C*, *MyOD* and *MyOG* was regulated	−	+	Slow muscle → fast muscle	[276,277,278]
	*PPARγ*	Phosphorylation of CAMKII and p38 MAPK promotes the expression of *MEF2* and *PGC-1αZ*	+	/	/	[280]
	*FHL3*	Inhibition of YY1 combined with MyHC-IIB	−	+	/	[281]
	*Prox1*	The trend of expression in porcine skeletal muscle was the same as that of MyHC-I and opposite to that of MyHC-IIB	+	−	+*	[282]
*Gallus gallus*	*SOX6*	Activation of *MEF2C* and inhibition of *Nfix*	+	/	/	[284]
	*ANKRD2*	Preferentially expressed in red slow muscle fibers of chickens	+	/	+*	[285]
	*Myoz3*	The PPAR signaling pathway regulates the conversion of muscle fibers and promotes the expression of the fast muscle marker gene *MYH1F*	−	+	+*	[287,288]
	*MyHC-IIA*	The cis-element interacts with E47 protein	+	−	+*	[289]
*Bos taurus*	*MSTN*	After knockout, *MyoD* was upregulated and *MEF2C* was downregulated	−	+	Slow muscle → fast muscle	[292]
	*F94L*	/	−	+	/	[293]
	*ADAM12*	Through phosphorylation and glycosylation	+	−	Fast muscle → slow muscle	[294]
	*ACTN3*	/	−	+	/	[295]
*Ovis aries*	*RIP140* *PGC-1β*	β-agonists upregulate *RIP140* and downregulate *PGC-1β* through the PPARδ signaling pathway	−	+	Slow muscle → fast muscle	[196]
	*RTL1* *DLK1*	Directly targeting the upregulation of *DNTTIP1* and *PDE4*, promoting the upregulation of *MYH4* and the downregulation of *MYH7*	−	+	/	[297]

“+” positive regulation; “−” negative regulation; “/” no regulation; “+*” The regulation of muscle fiber type is related to this process, but the direction of regulation has not yet been determined.

**Table 6:** Genes and non-coding RNAs that regulate the types of muscle fiber through signaling pathways.

Species	Signaling Pathways	Regulation of the Types of Muscle Fibers			Reference
		Slow Muscle	Fast Muscle	Transformation of Muscle Fibers	
*Sus scrofa*	Akirin2 inhibits the expression of *NFATc1*, *PGC-1α*, *MEF2C* and *MCIP1* through the Calcineurin/NFATc1 signaling pathway	−	+	Slow muscle → fast muscle	[298]
	Ferulic acid positively regulates the expression of mitochondrial transcription genes through the Sirt1/AMPK/PGC-1α signaling pathway	+	−	Fast muscle → slow muscle	[299]
	*FNDC5* directly positively regulates the expression of MyHC-IIA mRNA through the PGC-1α/FNDC5 signaling axis	/	+	/	[300]
	*FOXO1* regulates the type of muscle fiber through the PI3K/PKB and MAPK/ERK signaling pathways and the calcineurin/NFAT signaling cascade	−	+	Slow muscle → fast muscle	[301]
	Leucine reduces protein levels of its downstream transcription factor *FoxO1* by activating the Akt/FoxO1 signaling pathway	+	−	Fast muscle → slow muscle	[234]
	Fatty acid metabolism, degradation, and elongation pathways may be related to the formation of types of porcine skeletal muscle fiber	/	/	+*	[283]
*Gallus gallus*	IGF-1 affects protein synthesis in the pectoral muscle of poultry through the AKT/S6 signaling pathway, and IGF-1 is coordinated with the mRNA of the MSTN gene	−	+	+*	[302]
	The Hedgehog signaling pathway and calcium signaling pathway are key pathways that promote the formation of slow muscle fibers	+	/	/	[304]
*Bos taurus*	Resveratrol increases the expression of MyHC-IIA and MyHC-I proteins and decreases the expression of MyHC-IIB and MyHC-IIX proteins through the AMPK/SIRT1/PGC-1α signaling pathway	+	−	Fast muscle → slow muscle	[274]
	Pueraria may regulate the type of muscle fibers through the AMPK/PGC-1α/Nrf1 signaling pathway	+	−	Fast muscle → slow muscle	[305]
	ATRA upregulates the expression of MyHC-I-related genes through the PPARδ signaling pathway	+	−	Fast muscle → slow muscle	[306]
*Ovis aries*	Vitamin A stimulates mitochondrial biogenesis by activating the p38 MAPK/PGC-1α signaling pathway, inducing slow muscle fibers and inhibiting fast muscle fibers	+	−	/	[307]
	The AMPK/PGC-1α signaling pathway is involved in the conversion of Type II muscle fibers to Type I muscle fibers in grazing sheep	+	−	Fast muscle → slow muscle	[308]
	*PARK7* and *PDE4D* are potential targets of *DLK1* by activating the cAMP signaling pathway and the PI3K/AKT pathway, respectively	−	+	Slow muscle → fast muscle	[297]
*Bos mutus*	*MYH7*, *TNNT1*, *TPM3*, and *MYL3* regulate the distribution of muscle fibers mainly through the PPAR signaling pathway and the PI3K/Akt signaling pathway	/	/	+*	[126]

“+” positive regulation; “−” negative regulation; “/” no regulation; “+*” The regulation of muscle fiber type is related to this process, but the direction of regulation has not yet been determined.

## Data Availability

Data are contained within the article.
